# Analysis of variation of amplitudes in cell cycle gene expression

**DOI:** 10.1186/1742-4682-2-46

**Published:** 2005-11-11

**Authors:** Delong Liu, Kevin W Gaido, Russ Wolfinger

**Affiliations:** 1 CIIT Ceters for Health Research, 6 Davis Drive, Research Triangle Park, NC 27709, USA; 2 The SAS Institute Inc., SAS Campus Drive, Cary, NC 27513, USA

## Abstract

**Background:**

Variation in gene expression among cells in a population is often considered as noise produced from gene transcription and post-transcription processes and experimental artifacts. Most studies on noise in gene expression have emphasized a few well-characterized genes and proteins. We investigated whether different cell-arresting methods have impacts on the maximum expression levels (amplitudes) of a cell cycle related gene.

**Results:**

By introducing random noise, modeled by a von Mises distribution, to the phase angle in a sinusoidal model in a cell population, we derived a relationship between amplitude and the distribution of noise in maximum transcription time (phase). We applied our analysis to Whitfield's HeLa cell cycle data. Our analysis suggests that among 47 cell cycle related genes common to the 2^nd ^experiment (thymidine-thymidine method) and the 4^th ^experiment (thymidine-nocodazole method): (*i*) the amplitudes of CDC6 and PCNA, which are expressed during G1/S phase, are smaller in the 2^nd ^experiment than in the 4^th^, while the amplitude of CDC20, which is expressed during G2/M phase, is smaller in the 4^th ^experiment; and (*ii*) the two cell-arresting methods had little impact on the amplitudes of the other 43 genes in the 2^nd ^and 4^th ^experiments.

**Conclusion:**

Our analysis suggests that procedures that arrest cells in different stages of the cell cycle differentially affect expression of some cell cycle related genes once the cells are released from arrest. The impact of the cell-arresting method on expression of a cell cycle related gene can be quantitatively estimated from the ratio of two estimated amplitudes in two experiments. The ratio can be used to gauge the variation in the phase/peak expression time distribution involved in stochastic transcription and post-transcriptional processes for the gene. Further investigations are needed using normal, unperturbed and synchronized HeLa cells as a reference to compare how many cell cycle related genes are directly and indirectly affected by various cell-arresting methods.

## Introduction

Variation in gene expression is often considered as noise or uncertainty arising from experimental artifacts and biological variability. Various studies of noise in gene expression have focused on different scales, ranging from a single gene [1] to a single cell [2,3] to a cell population [4-9]. These studies have greatly helped us understand the effects of stochastic noise in gene expression and gene regulation in various model organisms. In a similar spirit, we were interested in the effects of different cell-arresting methods on the maximum expression levels (amplitudes) of some cell cycle related genes.

Various methods such as chemical induction and temperature shift have been used to arrest cells in genome-wide cell cycle studies [10-13]. Each method may have direct or indirect impacts on the synthesis or degradation of mRNAs from some genes after the interrupted cell cycle resumes. For example Whitfield et al. [11] used thymidine-thymidine (thy-thy) to arrest HeLa cells in G1/S phase and thymidine-nocodazole (thy-noc) to arrest them in G2/M phase. Intuitively, the synthesis or degradation of some mRNAs in G1/S phase and G2/M may be differentially affected by thy-thy and thy-noc arrests, respectively.

Measurements of the intensities of gene expression from microarray experiments are subject to two main sources of variation: (*i*) technical variability including bioassay preparation, dye-effect and hybridization on chips, (*ii*) and biological variability including variation in activation of transcription from cell to cell in a population after release from cell cycle arrest. Another implicit feature of microarray data is that gene expression is an average value over a cell population rather than in a single cell. In general, it is difficult to separate these two sources of variation for expression of a gene under given experimental conditions unless multiple repeated measurements are made over time and some prior knowledge of the expression of this gene is available. Periodic expression of some genes may be a good model for examining the effects of various cell-arresting methods on the transcription of known genes during cell cycle experiments.

**Figure 1:**
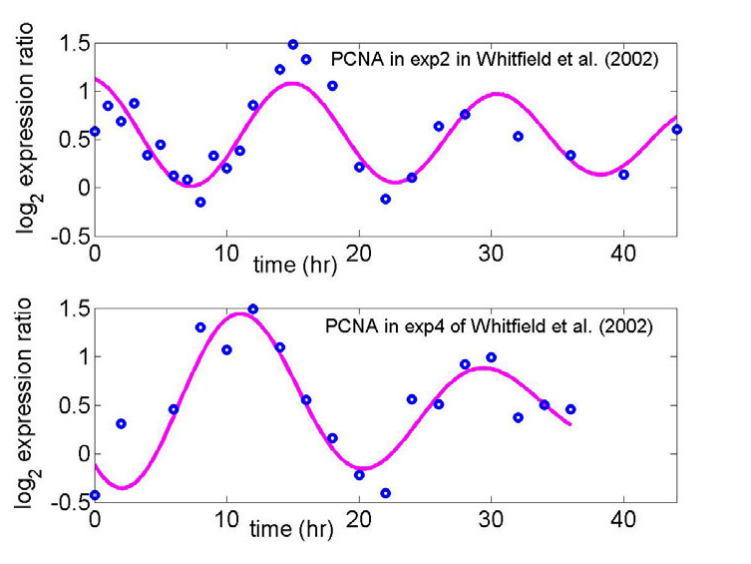
Log_2 _expression ratio for *PCNA*, a known G1/S phase gene, in thymidine-thymidine (exp2) arrest and thymidine-nocodazole arrest (exp4) studies. The solid line ('__') is the fit, which is estimated from the random-periods model (1), to the data ('o') from Whitfield et al. (2002).

**Figure 2:**
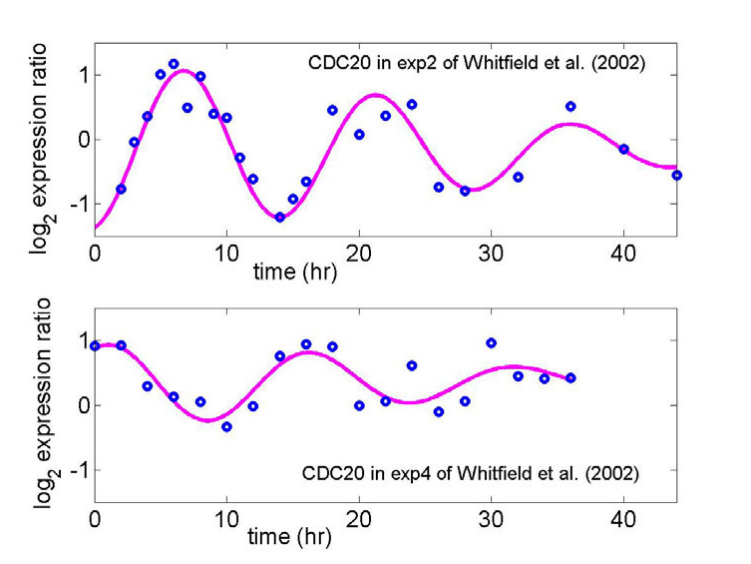
Log_2 _expression ratio for *CDC20*, a known G2/M phase gene, in thymidine-thymidine (exp2) arrest and thymidine-nocodazole arrest (exp4) studies. The solid line ('__') is the fit, which is estimated from the random-periods model (1), to the data ('o') from Whitfield et al. (2002).

Some advantages of using cell cycle related gene expression to probe the variation in maximum expression level due to different cell-arresting methods are: (*i*) cells can be synchronized to some extent so that variation of expression from cell to cell can be reduced; (*ii*) the expression profiles of some known cell cycle related genes such as PCNA and CDC20 (Figures 1 and 2) have been well characterized as sinusoidal waveforms over multiple cycles in different model organisms [10-13]. This makes it relatively easy to distinguish biological variation from technical variation, which produces random or transient fluctuations around a sinusoidal profile over time.

Amplitude, period and phase angle define the dynamics of a sinusoidal profile. In cell cycle or circadian rhythm studies, the phase angle, or time of maximum expression of a cycling gene, has been a primary focus because it reflects the gene's biological role [10-15]. However, the biological implications of amplitudes of cycling genes, referred to as the maximum expression level in one cycle, have not been explored in any previous microarray study of cell cycle or circadian cycle gene expression [10-15]. This might be due to the impression that gene expression from high-throughput data is noisy and therefore not reliable. Alternatively, it may be because no control (reference) mRNA was used across the experiments. When the expression of a cycling gene is measured across multiple time points in cell cycle modeled by a sinusoidal profile, its amplitude can be estimated with reasonable accuracy [16]. When a common reference mRNA is used in cell cycle experiments [11], the estimated amplitudes of the same cycling genes should be comparable across experiments. In addition to phases, changes in amplitude may reveal effects of cell-arrest methods on the expression of some cell cycle related genes.

In a single cell, the amplitude and phase of a cell cycle related gene are considered two independent parameters in a sinusoidal model. Within a cell population, however, variation in amplitude may be dependent on variation in phase angle for some genes of this kind when the cells are stressed at different stages of the cycle. The linking of amplitude to phase variability is similar to Winfree's suggestion about the connection: "Thirty-four years later the situation is beginning to change. It is at least widely recognized now that 'phase' is just one aspect of the circadian clock's 'state,' needing supplementation by at least 'amplitude' (possibly a measure of cell-population phase scatter) before experiments can be designed and interpreted with confidence" [17].

In this paper, we first illustrate how variation in amplitude depends on the distribution of phase angles of a cell cycle related gene in a cell population. We then analyze the effects of two different cell-arresting methods on some known cell cycle related genes expressed in G1/S and G2/M phases, using public cell cycle gene expression datasets.

## Methodologies

Three parameters are commonly used for modeling the time-course of expression, *y*_*g*_(*t*), of a cell cycle related gene *g *over time *t*: amplitude, which we denote as *K*_*g*_; duration of cycle (period), *T*; and phase angle, *φ*_*g*_, which is the time in the cycle when the gene is maximally activated; i.e. *y*_*g*_(*t*) = *f*(*t*; *K*_*g*_, *T*, *φ*_*g*_). In our previous cell cycle related gene expression studies [16], we introduced a variance parameter *σ *to *y*_*g*_(*t*) for modeling attenuation of the amplitude of gene *g *over time, leading to the following random-periods model (RPM):



where the integral averages the expression level across cells and *z *is assumed to be distributed as standard Gaussian. The linear terms, *a*_*g *_and *b*_*g*_, give the background gene expression. This model approximated the pattern of cycling, with its attenuation across time, when it was applied to a set of known cell cycle related genes [16].

Here, we introduce random noise, *ε*, to the phase of gene expression in a cell population into model (1). The expectation, *E*[ ], of the periodic term, which we call *c*_*g*_(t) in (1) for gene *g*, is



where *ε *is von Mises distributed with concentration parameter *κ *and mean direction 0, and *z *is, as before, normally distributed with mean 0 and variance 1. *K*_*gmax *_is the amplitude when *ε *= 0, i.e. no variation in phase/peak expression time for gene *g *in a population of perfectly synchronized cells. The expectation of *c*_*g*_(*t*) in (2), *E*⌊*c*_*g*_(*t*)⌋, can be expanded as



If the random variables *z *and *ε *are independent, we obtain the simplified expression



Since  for the random variable *ε *with a von Mises distribution, we obtain



Therefore, the amplitude *K*_*g *_in model (1) is the product of two terms, *K*_*g *max _and *E*[cos(*ε*)] in (3). *E*[cos(*ε*)] can be considered a measure of the variability in phase across cells in a given experiment. When the duration of the cell cycle is highly variable, as when *σ *is large in model (1), one might expect a corresponding attenuation of the amplitude over time.

Since it is difficult to estimate both the amplitude *K*_*g *max _and the term *E*[cos(*ε*)] directly from (3), we propose instead to compare the amplitude parameters in two independent experiments under the same protocol for *g *gene, by taking the ratio



where, , *κ*_*g *_is the concentration parameter of *ε *with a von Mises distribution [18], and *K*_*1g *_and *K*_*2g *_are the maximum expressions of gene *g *in experiments 1 and 2, respectively, when the phases or peak expression times for *g *in a cell population are perfectly synchronized. We have 0 ≤ *E*(cos(*ε*)) ≤ 1 as the concentration parameter *κ*_*g *_→ ∞, the variance goes to 0 and *E*[cos(*ε*)] = 1; and as *κ*_*g *_= 0, *E*[cos(*ε*)] = 0.

Provided that *K*_1*g *_= *K*_2*g*_, we reduce the ratio in (4) to



Equation (5) implies that the ratio between the amplitude parameters of periodic expression of gene *g *in experiments 1 and 2 can be represented by the ratio of the mean noise variation, which has von Mises distributions in both experiments. When *κ*_1 _>*κ*_2_, *E*[*c*_1*g*_(*t*)]_max _>*E*[*c*_2*g*_(*t*)]_max_. In biological terms, the concentration parameter, *κ*, reflects the distribution of phases or peak expression times for a gene within a cell population. Therefore, we can use the ratio of estimated amplitudes from RPM (1) to examine the relative variability in phase/peak expression time for gene *g *in two cell cycle experiments.

**Figure 3:**
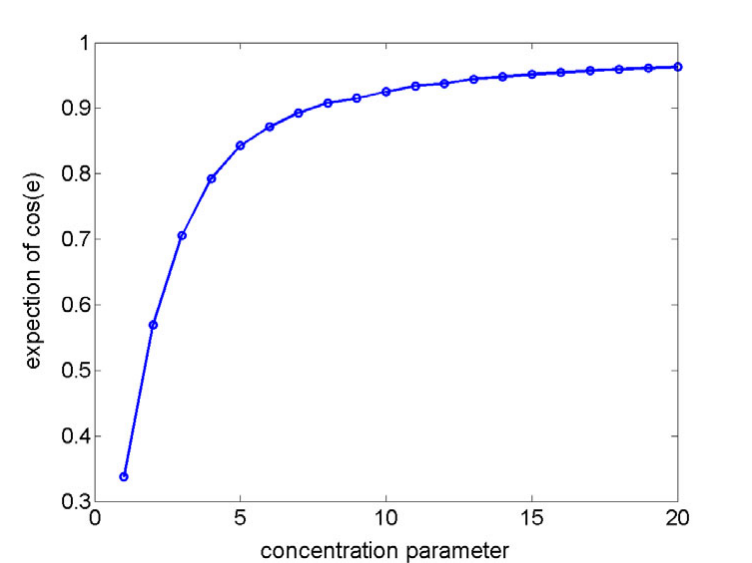
Plot of concentration parameter *κ *vs. expectation of cos(*ε*), where *ε *is von Mises distributed with zero mean direction and concentration *κ*, i.e., *ε *~ VM(*κ*,0).

To get a sense of how the ratios of estimated amplitude in (5) vary with *κ*, we calculated numerical values of *E*[cos(*ε*)] for the random variable *ε *with *μ *and *κ *= 1, 2, 3, ..., 20, and plotted *κ *vs. *E*[cos(*ε*)] in Figure 3. For *κ *= 1, 2, 3, 4, 5, *E*[cos(*ε*)] = 0.33, 0.57, 0.71, 0.79, 0.84, respectively. For example, for *κ *= 2 and 5, the ratio in (5) is 0.57/0.84 = 0.68. Note that *E*[cos(*ε*)] increases sharply and monotonically from *κ *= 1 to *κ *= 5. Figure 3 suggests that, for a cycling gene in two experiments with relatively large differences in amplitude, the concentration parameters *κ *in the experiment with small estimated amplitude are relatively small and most likely to be in the range 1 ≤ *κ *≤ 5. Although we have no direct knowledge of the true value of *κ *for a cycling gene in any experiment, we can still use Figure 3 to interpret the variation in transcription of a given gene within a cell population in multiple experiments. For example, within a HeLa cell cycle period of 15 h, phases in the interval (-0.65, 0.65) radians, or peak gene expression times in the interval (-1.5, 1.5) h, are within 95% coverage of the von Mises distribution with concentration parameter *κ *= 10.

In the following two sections, we apply the concepts presented above to the variation in amplitude of a set of cycling genes common to two experiments, using the cell cycle gene expression data of Whitfield et al. [11]. Here, we are primarily interested in assessing the variability of amplitudes of cell cycle related genes commonly expressed in two experiments where cells were arrested by two different methods, and in identifying genes of which the amplitudes *K*_*g *_do change in two experiments if there is no systematic variation between any pair of experiments.

## Testing equality of amplitudes of a set of cycling gene in two experiments

Let  and  denote the estimated amplitude and the variance of the amplitude for the *g*^th ^gene in the *j*^th ^experiment, *g *= 1, ..., *n*, where *n *is the number of genes and *j *= *x*, *y*.  is estimated from the random-periods model in (1), and  from Wald's sandwich estimator within the random-periods model (1). Prior to testing the equality of amplitude of a cycling gene in two experiments, we need to check whether there is a systematic variation in amplitude, which might be created during sample hybridization. For a set of *n *genes between two experiments, *x *and *y*, we take the difference



and use the Wilcoxon signed rank test to test the null hypothesis: median Δ = 0. If the null hypothesis is rejected, we suspect that there may exist a systematic difference between  and  in experiments *x *and *y*. If we fail to reject the null, there may be no true difference, or the statistical test lacked sufficient power to detect a true difference (which is small compared to the estimated noise in the experiment). In this situation we explore the results further to identify how many  and  may be equal for *g *= 1, ..., *n *by checking whether zero is included in the confidence interval  at the level of *α*, where  and  are the estimated variances of  and . If , transcription of the gene *g *might not differ between the two experiments.

## Example

In our previous work [19], we studied the phase association of 47 cell cycle related genes common to the 2^nd^, 3^rd ^and 4^th ^experiments of Whitfield et al. [11]. In the present study, we use the same 47 genes commonly expressed in the 2^nd ^and 4^th ^experiments with 26 and 19 time points per gene, respectively. The amplitude, period, geometric standard deviation, phase angle and two parameters describing the linear background, denoted respectively by (), were estimated for each expression time-course experiment using the random-periods model (1) on log_2 _transformed data. The assumptions underlying the model appear reasonable for these data, although our conclusions are somewhat limited given the small sample size. Owing to the systematically smaller amplitudes of the 47 cell cycle related genes in the 3^rd ^experiment of Whitfield et al. [11], which were identified by the Wilcoxon signed rank test of (6), we excluded the 3^rd ^experiment from our comparison of amplitudes in this study. The estimated amplitudes s, and the variances of the s, *g *= 1, ..., 47, in the 2^nd ^and 4^th ^experiments are listed in Table 1.

**Table 1:** Estimated amplitudes ,  and variances var(), var() of the amplitudes in the 2^nd ^and 4^th ^experiments of Whitfield *et al*. (2002), respectively.

Assession	Gene Symbol	K_2	var(K_2)	K_4	var(K_4)	lower bound	upper bound	flag
AA088457		0.921	0.026	0.642	0.007	-0.637	0.076	1
AA458994	PMSCL1	0.832	0.018	0.576	0.019	-0.635	0.122	1
AA485454		0.772	0.029	0.743	0.043	-0.554	0.495	1
AA485454		0.772	0.029	0.743	0.043	-0.554	0.495	1
AA282935	MPHOSPH1	0.950	0.030	0.843	0.049	-0.658	0.444	1
N57722	MCM6	0.401	0.013	0.596	0.024	-0.180	0.570	1
AA485454		0.747	0.035	0.743	0.043	-0.551	0.542	1
AA485454		0.747	0.035	0.743	0.043	-0.551	0.542	1
R11407	STK15	1.672	0.049	1.821	0.050	-0.467	0.765	1
T66935	DKFZp762E1312	1.648	0.051	1.319	0.035	-0.903	0.245	1
AA452513	KNSL5	1.162	0.033	1.155	0.062	-0.609	0.595	1
AA157499	MAPK13	1.375	0.045	1.360	0.060	-0.650	0.620	1
AA430092	BUB1	1.083	0.033	1.003	0.085	-0.755	0.593	1
AA053556	MKI67	1.315	0.056	0.790	0.043	-1.144	0.095	1
R96941	C20orf129	1.217	0.076	1.444	0.022	-0.387	0.840	1
AA131908	FLJ10540	0.786	0.016	0.390	0.014	-0.738	-0.053	0
AA279990	TACC3	0.794	0.026	1.023	0.055	-0.329	0.786	1
AA464019	E2-EPF	0.760	0.018	0.987	0.077	-0.378	0.832	1
AA262211	KIAA0008	0.918	0.013	0.688	0.030	-0.635	0.176	1
AI053446		0.964	0.041	0.952	0.050	-0.605	0.581	1
AA620485	ANKT	0.871	0.021	1.150	0.036	-0.192	0.750	1
AA629262	PLK	1.621	0.019	1.510	0.042	-0.597	0.375	1
AA450264	PCNA	0.557	0.008	0.985	0.038	0.007	0.849	0
R06900	RAMP	1.055	0.033	1.322	0.045	-0.280	0.814	1
AA425120	CHAF1B	0.549	0.006	0.552	0.032	-0.378	0.383	1
AA430511	FLJ14642	0.922	0.028	0.859	0.045	-0.592	0.465	1
AA430511	FLJ14642	0.922	0.028	0.786	0.061	-0.721	0.449	1
AA620553	FEN1	0.484	0.010	0.516	0.013	-0.270	0.335	1
AA402431	CENPE	1.468	0.015	1.455	0.082	-0.624	0.599	1
AA608568	CCNA2	1.197	0.016	1.115	0.076	-0.677	0.513	1
W93120		0.584	0.019	1.210	0.091	-0.026	1.278	1
N63744	FLJ10468	1.602	0.021	1.146	0.067	-1.038	0.125	1
R22949		1.055	0.026	0.908	0.045	-0.670	0.377	1
H51719	ORC1L	0.607	0.013	0.469	0.021	-0.500	0.222	1
AA425404	FLJ10156	1.101	0.041	0.841	0.010	-0.702	0.182	1
H59203	CDC6	0.695	0.014	1.060	0.020	0.005	0.724	0
AA292964	CKS2	0.827	0.010	1.516	0.185	-0.177	1.553	1
AA099033	USP1	0.507	0.012	0.750	0.027	-0.145	0.630	1
AA598776	CDC20	1.258	0.017	0.619	0.031	-1.067	-0.212	0
AA676797	CCNF	1.617	0.045	1.141	0.048	-1.072	0.121	1
AA504625	KNSL1	1.222	0.026	0.806	0.033	-0.893	0.062	1
AA235662	FLJ14642	1.041	0.015	0.944	0.041	-0.559	0.365	1
H73329	C20orf1	1.017	0.018	1.255	0.066	-0.330	0.807	1
AA421171	NUF2R	0.982	0.018	1.021	0.049	-0.467	0.546	1
T54121	CCNE1	1.155	0.045	1.144	0.052	-0.623	0.600	1
AA010065	CKS2	0.919	0.006	1.267	0.031	-0.028	0.724	1

Note that the accession numbers and the gene symbols were taken from the dataset of Whitfield *et al*. (2002). The genes with value 0 in the flag column indicate that the amplitudes are not same in the 2^nd ^and 4^th ^experiments.

## Results

**Figure 4:**
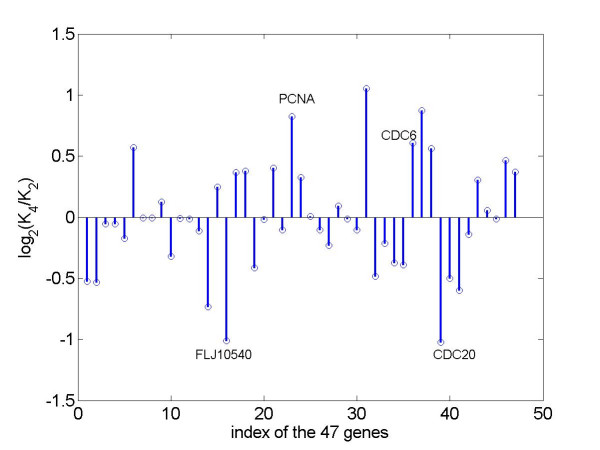
Plot of ratio of the amplitudes of 47 cell cycle transcripts in exp4 vs. exp2 (Whitfield *et al*., 2002).

The *p*-value from the Wilcoxon signed rank test on the median Δ = 0 in (6) at the level of *α *= 0.05 is 0.56, suggesting that the median amplitudes in exp2 and exp4 are similar. Therefore, we can directly compare the estimated amplitudes for each of the 47 genes in the two experiments. The log_2 _ratios of amplitudes in exp4 over exp2 are plotted in Figure 4. By comparing the amplitudes of the 47 cycling transcripts in these two experiments, we found that the 95% confidence intervals (*z*_*α*/2 _= 1.96, *σ *= 0.05) for the genes FLJ10540, PCNA, CDC6 and CDC20 did not include zero, suggesting that the estimated amplitudes for these four genes in exp2 and exp4 of Whitfield et al. [11] might be affected by thy-thy arrest in exp2 and thy-noc arrest in exp4. This was not true of the estimated amplitudes of the other 43 genes (Table 1). Note that the amplitudes of CDC6 and PCNA, which are expressed in the G1/S phase, were reduced almost to half in the thy-thy (S phase arrest) experiment relative to thy-noc (M phase arrest) experiment; the amplitude of CDC20, which is expressed in the G2/M phase, was reduced in the thy-noc experiment to half that in the thy-thy experiment.

## Discussion

In this paper, we have analyzed the effect of the scattering of phase angles of a cell cycle related gene in a cell population on the amplitude of expression of this gene. Our analysis suggests that variation in amplitude for such a gene between two experiments depends on the variation of phase distribution in a population of cells. We illustrated our analysis by comparing the amplitudes of 47 cell cycle related genes in the 2^nd ^and 4^th ^experiments of Whitfield et al. [11], where two different methods were used that resulted in cells being arrested at different stages of the cycle. The amplitudes of 43 of the 47 genes were not significantly affected by the differences in cell-arresting methods. Among the 4 genes that were differentially affected, the amplitudes of the G1/S phase genes CDC 6 and PCNA were smaller in the thy-thy (S phase arrest) experiment 2, while the amplitude of G2/M gene CDC20 was smaller in the thy-noc (M phase arrest) experiment 4 of Whitfield et al. [11]. These results suggest that thy-thy and thy-noc affected the maximum expression levels of some G1/S and G2/M phase genes differentially. It appears plausible that the thy-thy arresting method might completely prevent expression of some G1/S phase genes. Some of these genes could be recovered from the gene list of the 4^th ^experiment using the thy-noc method.

Our results suggest that thy-thy interrupts PCNA and CDC6 mRNA synthesis in S phase arrest, and thy-noc interrupts CDC20 and FLJ10540 mRNA synthesis in G2/M arrest. After the cells are released, synthesis of the mRNAs for some affected genes resumes but with large variation in pace across cells. In other words, the phase distributions of PCNA and CDC6 in the cell population of exp2 are more spread out during the G1/S phase; and the phase distributions of FLJ10540 and CDC20 in the cell population of exp4 are more spread out in the G2/M phase. For example, the ratio between the two amplitudes of CDC20 in exp4 vs. exp2 is about 0.5. According to the ratio defined in (5), we could infer that the upper bound for the concentration parameter  of von Mises for CDC20 in exp4 is less than 2.5, provided the  for CDC20 in exp2 is very large, e.g. >20. The significant difference between the two distributions with  = 2 and 10 is illustrated graphically in Figure A in the Appendix.

Our results show that some cell cycle related genes may be more responsive or sensitive than others to changes in the environment, e.g. cell-arresting chemicals, temperature shift, etc. Raser and O'Shea [8] suggested that noise intrinsic to eukaryotic gene expression is gene-specific, and Fraser *et al*. [9] suggested that the production of essential and complex-forming proteins involves lower levels of noise than does the production of most other genes. Our findings indicate that the 43 cell cycle related genes with unaltered amplitudes in exp2 and exp4 of Whitfield et al. [11] may be essential to the HeLa cell cycle, and thus less sensitive to perturbation by stress or chemicals. However, CDC6 and CDC20, which are important to the yeast cell cycle [20], were expressed at significantly different amplitudes in the HeLa cell cycle. Further studies are needed to investigate whether some essential cell cycle genes such as CDC6 and CDC20 are cell type specific in response to chemicals.

The amplitude, phase angle and period estimated from (1) for genes from the microarray data are characteristic of cell populations rather than a single cell. Conventionally, amplitude and phase angle are considered independent parameters in a sinusoidal model. However, in microarray studies, where the measured periodic expression for a cell cycle related gene is averaged over a cell population (>10^6 ^cells), a phase change in the concentration of von Mises distribution for a gene can contribute to a change in amplitude. Note that our analysis partially addresses Winfree's concern about whether amplitude should be considered as additional information to phase in studies of circadian rhythms [17].

The detection of cell cycle related genes with significantly different amplitudes between exp2 and exp4 of Whitfield et al. [11] depends on: (*i*) approximation of the true distribution of amplitudes of *K*_*gx *_and *K*_*gy*_, *g *= 1, ..., 47 by a normal distribution; (*ii*) the design of exp2 and exp4, including number of time points per gene. While these assumptions appear tenable for these data, a more comprehensive analysis of other relevant cell cycle gene expression studies is needed for more definitive conclusions about their validity. The four genes currently identified all have an estimated 1.5 fold change, and with the current sample size, the power to detect such a change is only around 50%. If the number of time points in exp2 and exp4 were larger (e.g. 47 in exp3 of Whitfield et al. [11]), the power for detecting amplitudes with less than 2-fold change would be increased.

One often neglected but important factor in interpreting and analyzing cell cycle related gene expression data is the quality of synchrony of the cell culture. Currently there are no quantitative standards for measuring to what extent cells have been synchronized. The periodic patterns of the 47 genes were measured from stressed or perturbed cells in the 2^nd ^and 4^th ^experiments of Whitfield et al. [11]. Gene expression from normal, un-perturbed and synchronized HeLa cells obtained using the technologies proposed by Helmsteteter et al. [21] may serve as references for comparing the expression of these genes when mRNA synthesis is interrupted by different cell-arresting methods, e.g. temperature shift or chemical induction at various phases of the cell cycle. Good quality control of cell synchrony, as suggested in Cooper et al. [22], will provide a basis for microarray studies of cell cycle related genes. More quantitative measures of cell culture synchrony, and investigation of the impacts of cell culture with various degrees of synchrony on expression of some cell cycle related genes, are needed in future studies.

## Conclusion

The amplitudes of some cell cycle related genes were used to measure the effects of two different cell-arresting methods on gene expression. Some genes with periodic expression patterns can be used as models to probe the effects of different cell-arresting methods on expression of these genes, which can be quantitatively described in terms of amplitude and phase. The ratio between the amplitudes estimated in two experiments for a cell cycle related gene can be used to gauge the variation of the phase/peak expression time distribution involved in stochastic transcriptional and post-transcriptional processes for the gene in a cell population. Further investigations are needed using normal, unperturbed and synchronized HeLa cells as a reference for comparing how many cell cycle related genes are directly and indirectly affected by various cell-arresting methods.

## Competing interests

The author(s) declare that they have no competing interests.

## Authors' contributions

DL conceived of the study, performed the analysis and drafted the manuscript. KWG and RW participated in the draft of the manuscript. All authors read and approved the final manuscript.

## Supplementary Material

Additional file The addition file 'Appendix.doc' is inserted here. Click here for file
