# Supplementary material for: Analysis of variation of amplitudes in cell cycle gene expression
Source: Theor Biol Med Model. 2005 Nov 11;2:46. doi: 10.1186/1742-4682-2-46 (PMC1315354; doi:10.1186/1742-4682-2-46)
Supplement: Additional file — The addition file 'Appendix.doc' is inserted here. [file 1742-4682-2-46-S1.doc]

**Appendix**: **von Mises Distributions**

The von Mises distribution *VM*(*μ,κ*) has probability density function

$f(\varphi;\mu,\kappa)=\frac{1}{2\pi I_{o}(\kappa)}\exp(\kappa\cos(\varphi-\mu))$, (A1)

where *I_o_* denotes the modified Bessel function of the first kind and order 0, which can be defined by $I_{o}(\kappa)=\frac{1}{2\pi}\int_{0}^{2\pi} \exp(\kappa\cos(\varphi))\begin{matrix} d\varphi\end{matrix}$. The parameter *μ* is the mean direction; the parameter *κ* is known as the concentration parameter. For detailed information regarding von Mises distribution, refer to [18].


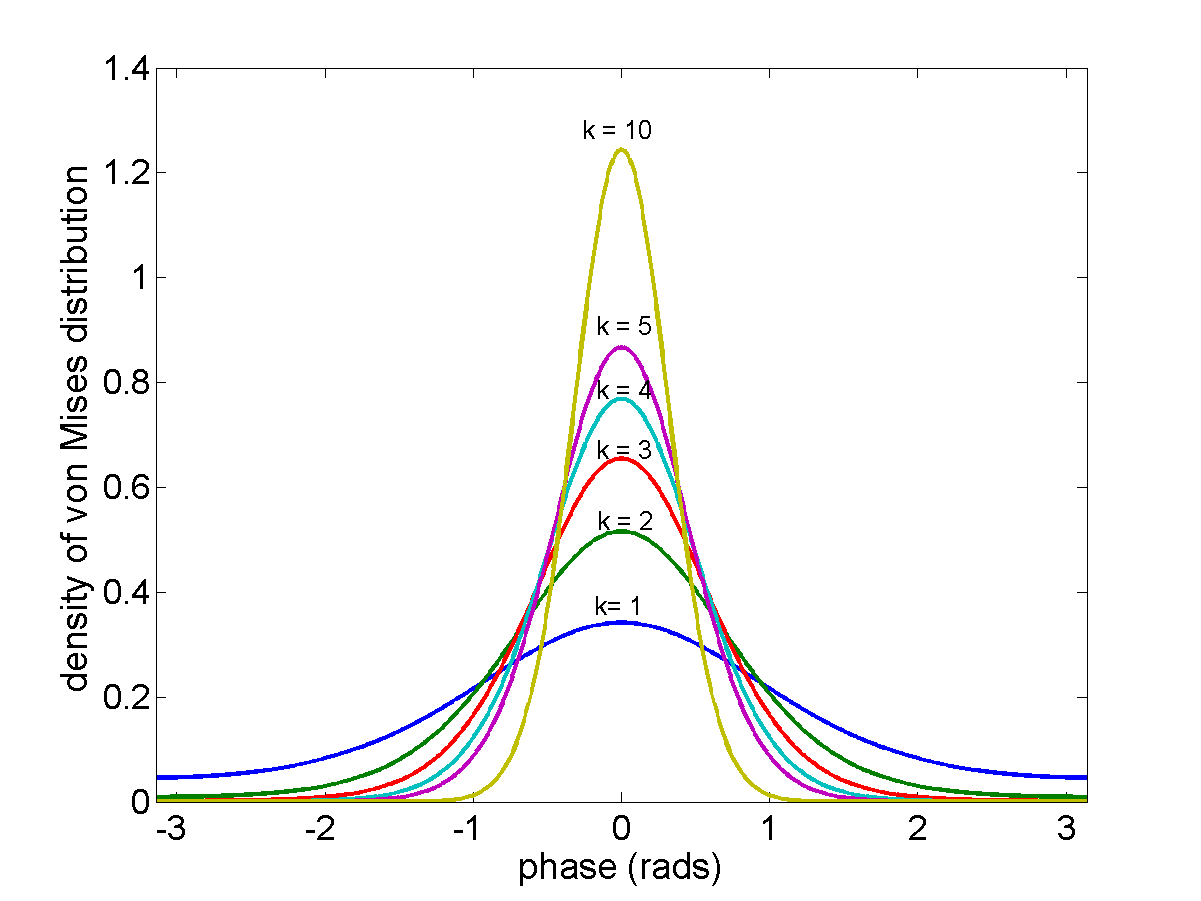


Figure A. Density of von Mises distribution *VM*(0, *κ*) for *κ* = 1, 2, 3, 4, 5, 10.
